# Optimization of production, purification and lyophilisation of cellobiose dehydrogenase by *Sclerotium rolfsii*

**DOI:** 10.1186/s12896-014-0097-5

**Published:** 2014-11-19

**Authors:** Christin Fischer, Annett Krause, Thomas Kleinschmidt

**Affiliations:** Anhalt University of Applied Sciences, Department of Applied Biosciences and Process Engineering, Bernburger Str. 55, 06366 Köthen, Germany

**Keywords:** Cellobiose dehydrogenase, CDH, *Sclerotium rolfsii*, Enzyme purification, Lyophilisation, Cryoprotection, Lactobionic acid

## Abstract

**Background:**

The enzyme cellobiose dehydrogenase (CDH) can be used to oxidize lactose to lactobionic acid. As *Sclerotium rolfsii* is known to be a good producer of CDH, the aim of this paper was to simplify its production and secondly to systematically study its purification aiming for a high yield. Two preservation methods (freezing and freeze-drying) and the influence of several protectants were investigated.

**Results:**

Production of cellobiose dehydrogenase was optimized leading to a more simplified medium composition. Purification of the enzyme was evaluated by determining breakthrough profiles on different ion exchange (IEX) and hydrophobic interaction (HIC) materials with regard to buffer composition. Highest purification with an acceptable loss during the capture step using IEX was obtained with a Q Sepharose XL medium and a 100 mM sodium acetate buffer at pH 4.5. Subsequent purification using hydrophobic interaction chromatography was done at 1.1 M ammonium sulfate concentration. Purification was moderate, yielding a specific activity of 11.9 U/mg (56% yield). However, as could be shown in a preliminary experiment, purity of the obtained enzyme solution was sufficient for its intended use to oxidize lactose to lactobionic acid. Various sugars and sugar alcohols were investigated to study their protective effect during lyophilisation and freezing at -20°C. Glucose and lactulose could be identified to have a high lyoprotective effect while loss of enzyme activity was high (77%) when using no additives.

**Conclusion:**

By simplifying the cultivation medium of *Sclerotium rolfsii*, the costs of cellobiose dehydrogenase production could be reduced. Simultaneously, CDH production was increased by 21%. The production of lactobionic acid from lactose is possible using partially purified and unpurified enzyme. Storage at -20°C using 50% (w/v) glycerol was considered to be most suited for preservation of the enzyme.

## Background

Cellobiose dehydrogenase (CDH, EC 1.1.99.18) is an extracellular enzyme produced by a number of wood-degrading fungi [1]. One of them is the plant pathogen *Sclerotium (Athelia) rolfsii* which is common in the tropics and subtropics and attacks mostly crops and vegetables. It is mainly producing cellulolytic enzymes in order to enter the host organism [2]. The biological function of CDH is controversial. For example, CDH increases the efficiency of cellulose degradation or reduces product inhibition of cellulases by oxidizing cellobiose to the corresponding lactone [1,3]. This theory was also confirmed several years later [4].

CDH is a monomeric protein consisting of a flavin and a heme domain. Both domains are connected by a protease-sensitive linker. When CDH is cleaved by proteases, it results in an active flavin domain and an inactive heme fragment. It is possible to distinguish between the holoenzyme and the flavin domain by using both one and two electron acceptors. Two electron acceptors like 2,6-dichlorophenol-indophenol (DCIP) can be reduced either directly at the flavin domain or via internal electron transfer at the heme domain. One electron acceptors like cytochrome c can only be used at the heme domain. Thus, they are used to detect only the intact enzyme.

**Figure 1:**
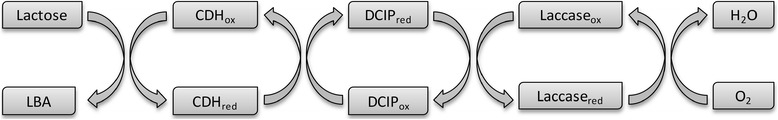
**Reaction scheme of enzymatic oxidation of lactose to lactobionic acid (LBA).** The used enzyme system consists of cellobiose dehydrogenase (CDH) and laccase with 2,6-dichlorophenol-indophenol (DCIP) as redox mediator.

As indicated by its name, cellobiose is the main substrate of the enzyme [5–9] indicated by a low Michaelis Menten constant. Other cellooligosaccharides are also favoured [10] but another common substrate is lactose. Figure 1 shows the reaction mechanism using DCIP as a redox mediator. The corresponding lactone is spontaneously oxidized to lactobionic acid which is rumored to have prebiotic effects [11]. Thus, an application in the food industry is a possible alternative to its current usage in biosensors for glucose ([12,13]) or lactose ([14–16]) and as a bleaching agent ([17–19]).

As *Sclerotium rolfsii* is known to be a good producer of CDH, the aim of this paper was to simplify its production and secondly to systematically study its purification aiming for a high yield. Two preservation methods (freezing and freeze-drying) and the influence of several protectants were investigated.

## Methods

### Organism and culture conditions

*Sclerotium rolfsii* strain CBS 191.62 was obtained from the Centraalbureau voor Schimmelcultures (Baarn, The Netherlands). The fungus was maintained on glucose-maltose Sabouraud agar plates, which were inoculated with a piece (diameter 1 cm) of overgrown agar and then incubated at 30°C for 5 to 7 days.

A medium propagated by Sachslehner et al. [2] was used in some studies. It contained 43 g/L α-Cellulose, 80 g/L peptone from meat, 2.5 g/L NH_4_NO_3_, 1.5 g/L MgSO_4_ x 7H_2_O, 1.2 g/L KH_2_PO_4_, 0.6 g/L KCl , and 0.3 ml/L trace element solution (1.0 g/L ZnSO_4_ x H_2_O, 0.3 g/L MnCl_2_ x 4H_2_O, 3.0 g/L H_3_BO_3_, 2.0 g/L CoCl_2_ x 6H_2_O, 0.1 g/L CuSO_4_ x 5H_2_O, 0.2 g/L NiCl_2_x 6H_2_O and 4.0 ml/L conc. H_2_SO_4_). The natural pH of the medium was 5.5. For optimizing the culture conditions of *Sclerotium rolfsii* with regard to maximum production of cellobiose dehydrogenase, start pH was varied (5.5, 5.0 and 4.0) by adding appropriate amounts of phosphoric acid. Additionally, a pH-stat method for a start pH value of 5.5 was carried out by measuring and readjusting the pH to 5.5 with 5 M NaOH on a daily basis.

Further, the composition of the medium was modified. The medium of Sachslehner et al. [2] was used as a reference. First, single salts were omitted from the medium described above to evaluate their influence on enzyme production. In a second series, a basal medium containing 43 g/L α-Cellulose, 80 g/L peptone from meat and 0.3 ml/L trace element solution was used. NH_4_NO_3_, MgSO_4_, KH_2_PO_4_, KCl or none of them were added (amounts as in the reference medium).

All experiments were carried out in 300 ml unbaffled Erlenmeyer flasks containing 100 ml culture medium. Flasks were inoculated with 2 agar plugs (diameter 1 cm) of a freshly grown culture and incubated at 30°C and 150 rpm until enzyme activity remained constant. Samples of 1 ml were withdrawn starting after 7 days of incubation and analyzed for enzyme activity.

### Enzyme purification

First, the mycelia were separated from the cultivation medium containing the CDH enzyme by filtration using a folded filter. This crude enzyme extract was desalted and concentrated to about one fifth of its initial volume using a Vivacell 250 filtration device (Sartorius AG, Germany) equipped with a PES membrane having a MWCO of 50 kDa. Within two dialysis steps and applying a pressure of 4 bar, conductivity was reduced from about 18 mS/cm to about 1 mS/cm indicating a successful salt removal. No enzyme loss was detected during this step.

Enzyme purification was carried out on an ÄKTA Prime Plus system (GE Healthcare) equipped with a 150 ml superloop for the application of large sample volumes. Different adsorbents for anion exchange (DEAE Sepharose FF as a weak and Q Sepharose XL as a strong ion exchanger, column volume: 5 ml, both by GE Healthcare) and hydrophobic interaction chromatography (Butyl-S FF, Butyl FF, Octyl FF, Phenyl HP, Phenyl FF (low sub), column volume: 1 ml, all by GE Healthcare) were tested to optimize purification procedure. With IEX, binding capacity was determined at various pH values (50 mM sodium acetate buffer) and buffer concentrations (20 – 200 mM sodium acetate, pH 4.5) by applying 110 ml of desalted and appropriately diluted crude enzyme extract (final concentration about 3 U/ml) onto the column. Fractions (2 ml) of the flow-through were collected and analyzed for CDH activity to determine a breakthrough profile. Also, elution conditions were optimized by analyzing the collected fractions for enzyme activity and protein content. Fractions having a higher specific activity than the applied sample were pooled. Ammonium sulfate precipitation was carried out by adding an appropriate amount of a 3.8 M solution to obtain the desired saturation before applying the sample to various HIC columns. Concentration and composition of the starting buffer were optimized as well.

### Preservation of the enzyme

Seven sugars and two sugar alcohols were tested for their ability to serve as lyo- or cryoprotectants for CDH. Therefore 1 ml of purified enzyme solution (containing 11 U/ml) and 0.5 ml of lyoprotectant solution (to give a final concentration of 10 μmol per unit CDH) were mixed and either frozen at -20°C or freeze dried in 1.5 ml Eppendorf tubes. Freeze-drying was carried out in an Alpha 1-4 freeze-dryer (Martin Christ Gefriertrocknungsanlagen GmbH, Germany). Plate temperature was set to -50°C and within 3 hours a product temperature of -28°C was obtained. Vacuum (0.005 mbar) was applied and plate temperature was set to 30°C. The process was stopped after 4 to 5 days when product temperature was positive. Samples were rehydrated with 1 ml water immediately after drying and analyzed for residual enzyme activity. Frozen samples were thawed after 60 and 160 hours and also assayed for remaining CDH activity.

### Enzyme activity assay

For measuring the enzyme concentration, the DCIP assay as propagated by Baminger et al. [20] was used. Therefore, 100 μl 300 mM lactose, 20 μl 200 mM NaF and 760 μl 100 mM sodium acetate buffer pH 4.0 were incubated at 30°C for at least 10 minutes before analysis. A solution of 3 mM DCIP (containing 10% v/v ethanol) was tempered separately. For analysis, 100 μl of DCIP solution were added to the lactose/NaF/buffer mixture and 20 μl appropriately diluted sample solution was added. After mixing the reduction of DCIP was measured at 520 nm every 5 seconds for 3 minutes. The extinction coefficient for DCIP at 520 nm and pH 4.0 was determined to be 6.9 mM^−1^ cm^−1^. One unit was defined as the amount of enzyme that reduces 1 μmol DCIP per minute under the described assay conditions.

### Protein determination

Protein content was determined according to the Bradford method [21]. Therefore 100 μl appropriately diluted sample solution were mixed with 1 ml of Bradford reagent (Roti®-Quant, Carl Roth GmbH, Germany) and incubated for 10 minutes at room temperature. Extinction was measured at 595 nm. Bovine serum albumin was used as a standard.

### Application of CDH for lactobionic acid synthesis

The partial purified enzyme as well as the crude enzyme extract were used in a 4.8% lactose solution to synthesise lactobionic acid. Enzyme substrate ratio was set to 70 DCIP-Units per gram lactose. DCIP (1 μmol/unit CDH) was used as a redox mediator and laccase from *Trametes versicolor* (Sigma Aldrich, Germany) was added in a 5-fold excess over CDH activity. Reaction was carried out in a water bath at 35°C and 200 rpm. Samples were analyzed for glucose, galactose, lactose and lactobionic acid using HPLC. The column was a Hi-Plex Na column (300 mm x 7.7 mm from Agilent Technologies Deutschland GmbH) which was used at 0.3 mLmin^−1^ (eluent 0.2% sodium azide in water) and 80°C.

## Results and discussion

### Influence of culture conditions on CDH yield

#### Influence of pH

**Figure 2:**
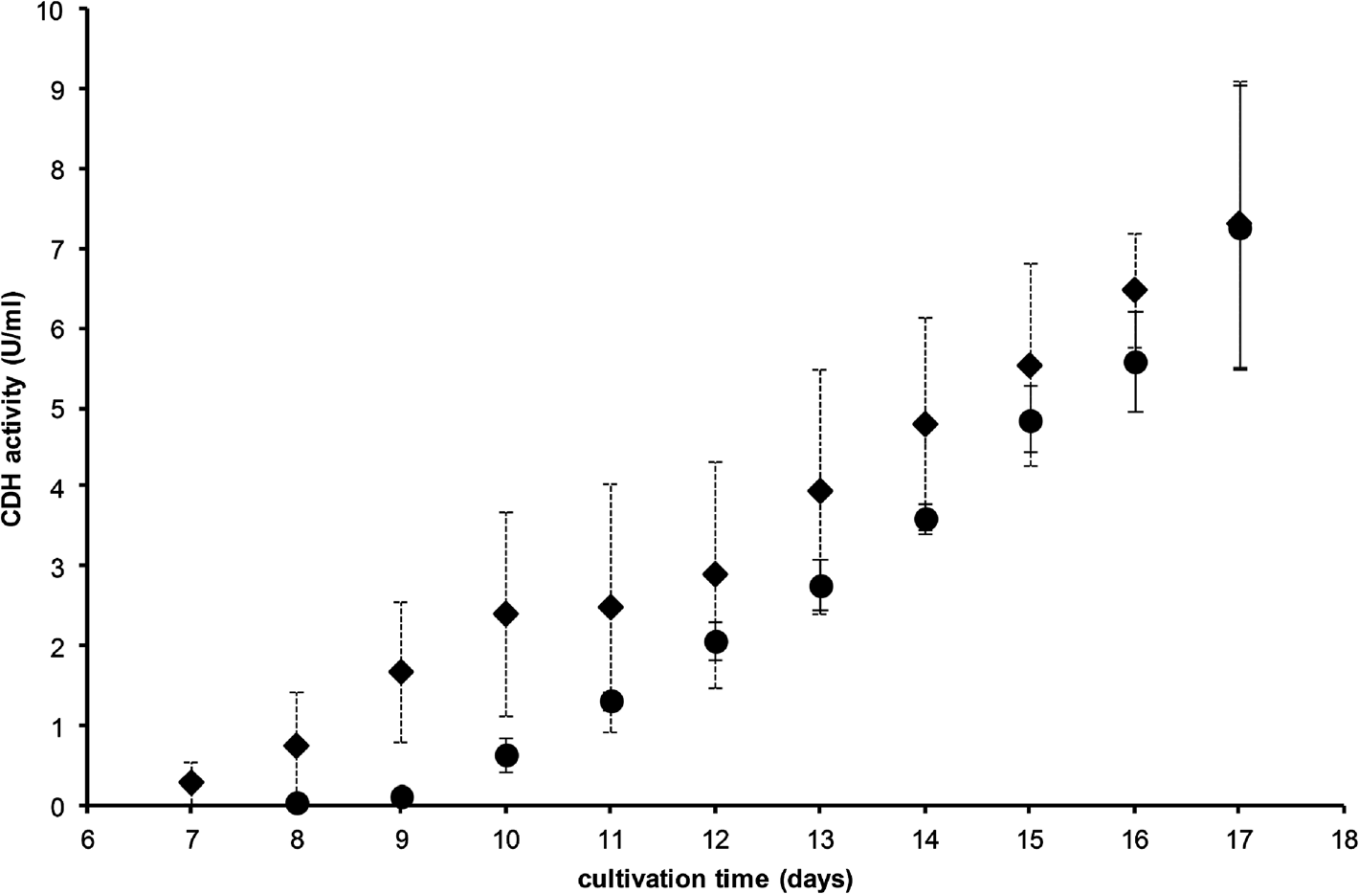
**Influence of start pH on CDH production by** ***S. rolfsii*** **.** pH 5.5 (♦, n = 18), pH 5.0 (●, n = 4).

Figure 2 shows the influence of the starting pH of the medium. At pH 5.0 CDH production begins noticeably at day 10, continues to rise straight and reaches a value of 7.3 U/ml after 17 days. With a slightly higher pH of 5.5 CDH production started a little earlier (day 7) and was significantly higher until day 10. Thenceforward the measured enzyme activity was a little higher compared to a starting pH of 5.0 but that difference was not significant. Also, after 17 days the produced enzyme amount was the same as with pH 5.0. Although cultivation at a starting pH of 5.5 seemed to be a little instable (as indicated by high standard deviations), this value was used for further studies as 5.5 is the natural pH of the medium and therefore no adjustment is necessary. There was only very little (0.1 U/ml) CDH production at a pH of 4.0 (data not shown). Therefore this pH is not suitable.

Since the pH of the culture medium shifts to lower levels during cultivation, it was assumed using a pH-stat method to cultivate at a constant pH value of 5.5 might further enhance CDH production. Therefore, an appropriate amount of 5 M NaOH was added on a daily basis. However, a maximum enzyme concentration of only 2.6 U/ml was reached after 13 days. It then slightly dropped down to 2.3 U/ml and remained on that level until the end of the experiment after 21 days. CDH production using cultivation without pH adjustment was 3 times higher at that time. Consequently, control of pH is not favorable.

#### Influence of medium composition

**Figure 3:**
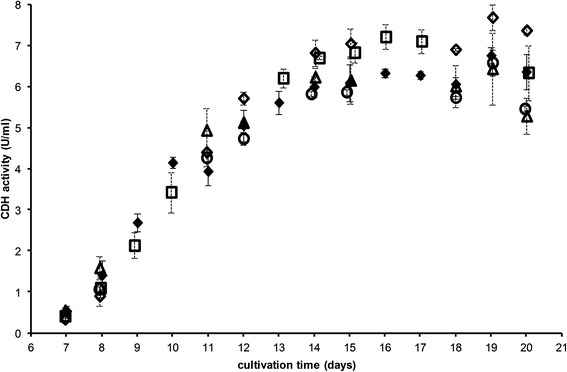
**CDH production by** ***S. rolfsii*** **.** reference medium (♦, n = 4), medium lacking individual salts (without NH_4_NO_3_ (◊)/KCl (Δ): n = 3, without KH_2_PO_4_ (□)/MgSO_4_ (○): n = 2).

Influence of medium composition was studied by omitting a particular salt in the medium proposed by Sachslehner et al. [2]. The latter was used as a reference medium. As can be seen from Figure 3, enzyme activity in the reference medium is constantly increasing from day 7 to 13 and then maintains at a nearly constant level from day 14 to 20.

KH_2_PO_4_ seems to have a negative impact on enzyme production as when omitting this salt, a significantly higher enzyme concentration could be reached as can be seen clearly from the data at days 14 to 17. Although, compared to the reference medium there seems to be a small decline in enzyme concentration with ongoing cultivation, so that at day 20 enzyme activity reached the same value (6.3 U/ml). The same can be conclude for NH_4_NO_3_ as there is significantly higher enzyme production from day 12 to 20 when this salt is omitted. In contrast to KH_2_PO_4_, enzyme concentration is enhanced until the end of the experiment resulting in about 15% higher CDH concentration compared to the reference medium. On the other hand, KCl and MgSO_4_ seem to have no impact on CDH production as when omitting these salts no significant difference in enzyme production was observed. However, there might be a tendency that CDH is depleted by the fungus if cultivation would be continued as indicated by considerably lower enzyme activity values at day 20.

**Figure 4:**
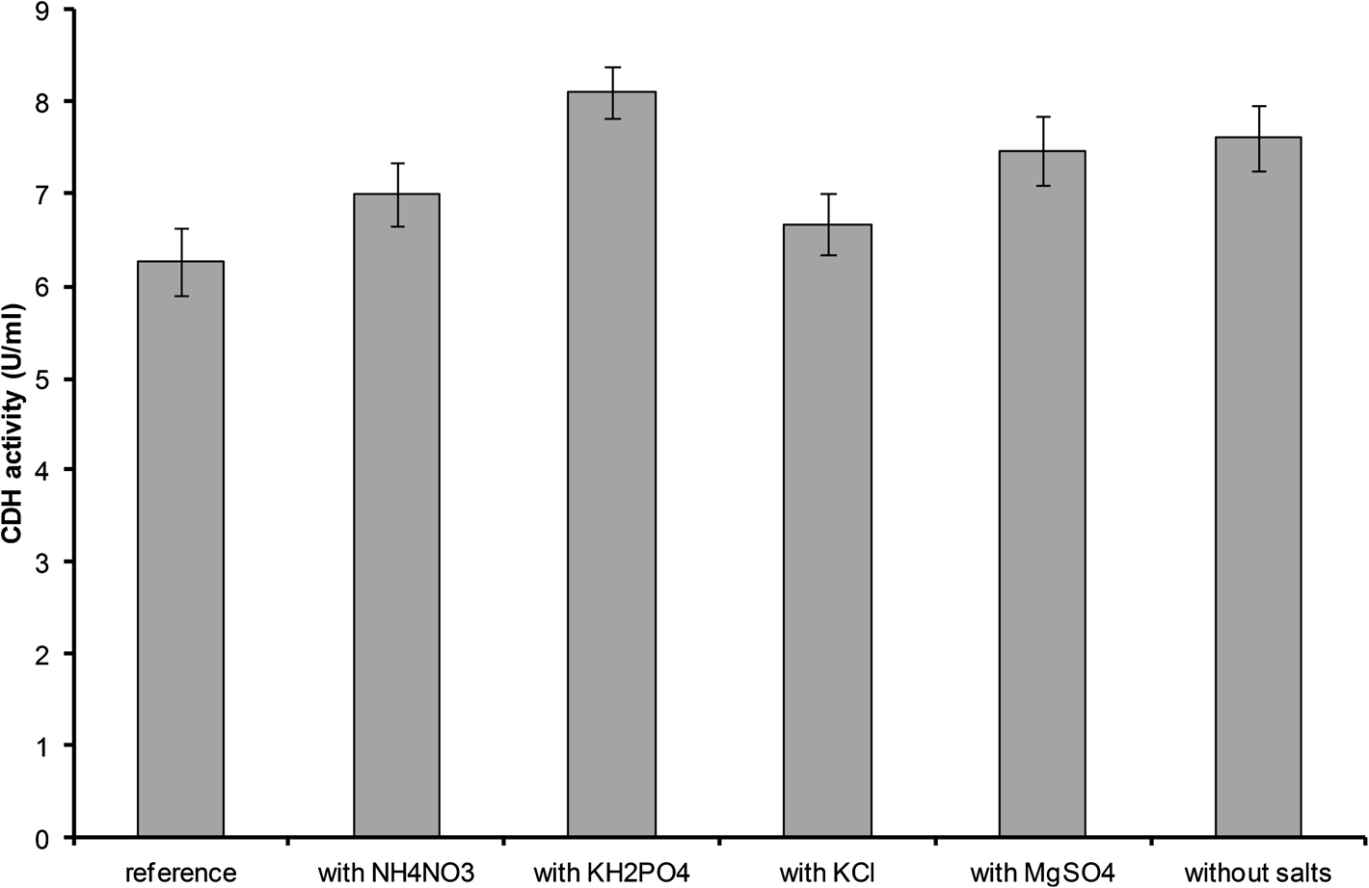
**CDH production by** ***S. rolfsii*** **after 19 days of cultivation in different culture media.** reference n = 6, NH_4_No_3_ n = 3, KH_2_PO_4_ n = 2, KCl n = 4, MgSO_4_ n = 5, without salts n = 5.

A second approach was used to gain more insight in the influence of each salt. At that none or only one salt at a time was added to a basal medium containing only α-cellulose, peptone from meat and trace element solution (amounts as in the reference medium). In all cases enzyme production by *S. rolfsii* started at the same time and reached a maximum at day 19. There were no significant differences in CDH production in the first 15 days but the influence of all salts became more pronounced later (data not shown). As can be seen in Figure 4, no matter which salt was added, CDH production was higher compared to the reference medium. The effect was only marginal with KCl and NH_4_NO_3_ but more distinct when adding KH_2_PO_4_ or MgSO_4_. This is in contrast to the results presented above which showed no effect of MgSO_4_ and a negative impact of KH_2_PO_4_. However, with the presence of other salts, the conditions were different and therefore the impact of the individual salts could play a diverse role. As was expected, adding none of the tested salts to the basal medium also resulted in significantly higher enzyme concentration. On day 19, 21% more enzyme was produced in the medium containing α-cellulose, peptone from meat and trace element solution than in the reference medium. CDH concentration began to decline afterwards so one has to be careful to choose the right harvesting time. Also, when additionally omitting the trace element solution (i.e. only cultivating the fungus in α-cellulose and meat peptone solution), a similar CDH production was observed (data not shown).

Using the same cultivation method (flasks containing 100 ml medium), Sachslehner et al. [2] obtained an enzyme activity of 3.6 U/ml after 13 days of cultivation. Ludwig and Haltrich [22] achieved an enzyme concentration of 4.1 U/ml after 14 days. In 2003 they reported about 7 U/ml in the same medium after 13 days [23]. With reducing peptone from meat to 20 g/L and adding 30 g/L leucine instead, 11 U/ml were obtained after 13 days. This good result could not be confirmed in the present study as enzyme production using this medium composition was very low (1.2 ± 0.9 U/ml after 16 days (n = 4)). The difference might be due to the usage of meat peptone from different suppliers therefore resulting in a different composition.

### Enzyme purification

#### Optimization of ion exchange chromatography

**Figure 5:**
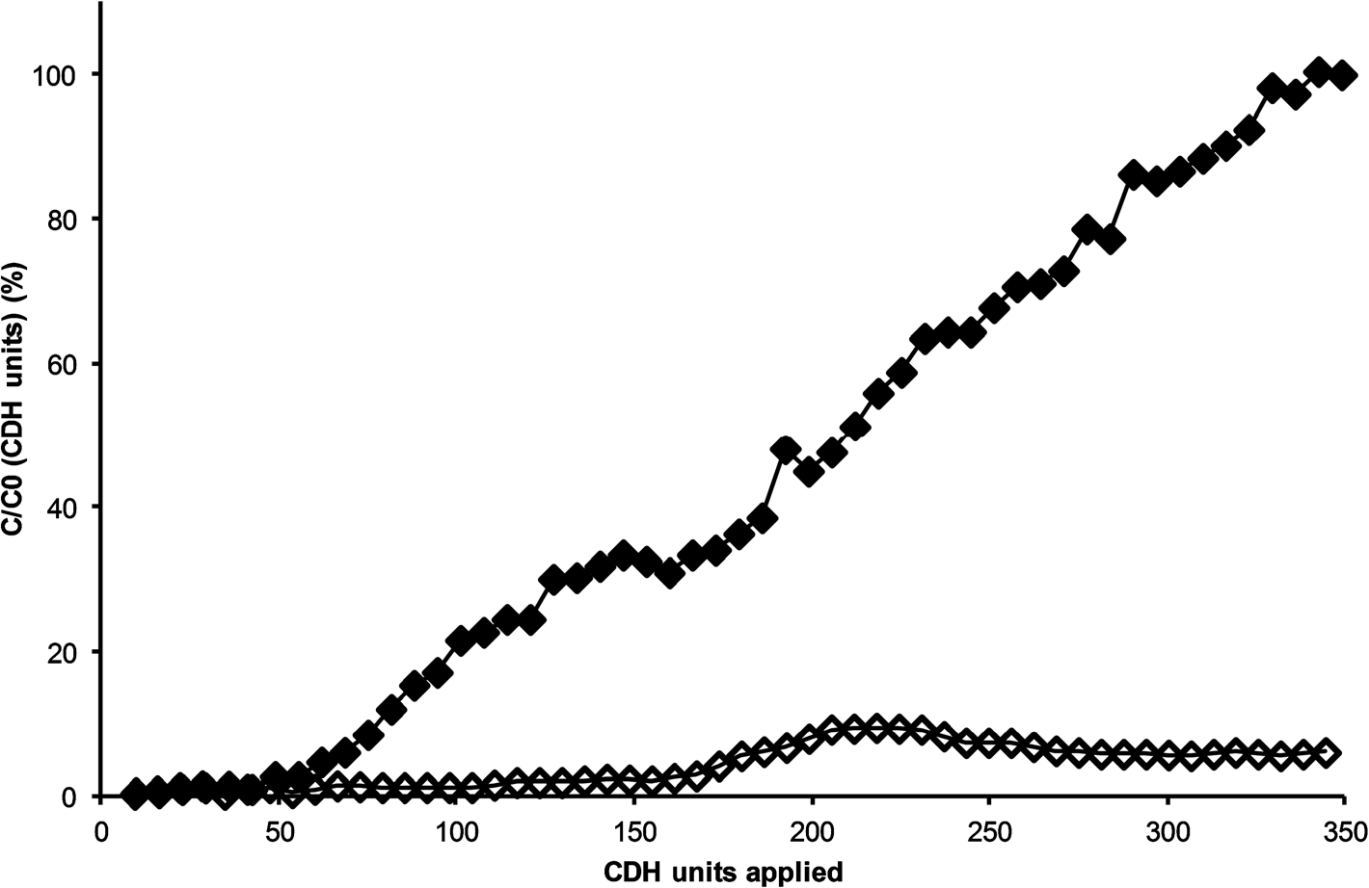
**Breakthrough curves for CDH at pH 5.0.** DEAE Sepharose FF (♦), Q Sepharose XL (◊).

To choose an appropriate anion exchange medium, the binding capacity of two materials was determined by applying 350 units onto the column and determining breakthrough curves. As can be seen from Figure 5, with the weak anion exchange medium (DEAE Sepharose FF) the breakthrough was negligible until about 50 units were applied. After that, the amount of unbound enzyme increases linearly thus reaching a value of c/c_0_ of 100% (meaning the sample leaves the column as it is, no more enzyme binds onto the column) after 350 units were applied. In contrast, with the strong anion exchanger (Q Sepharose XL) at the same pH of 5.0, the breakthrough profile always stays below 10% therefore indicating to be more suitable for CDH binding in general.

**Figure 6:**
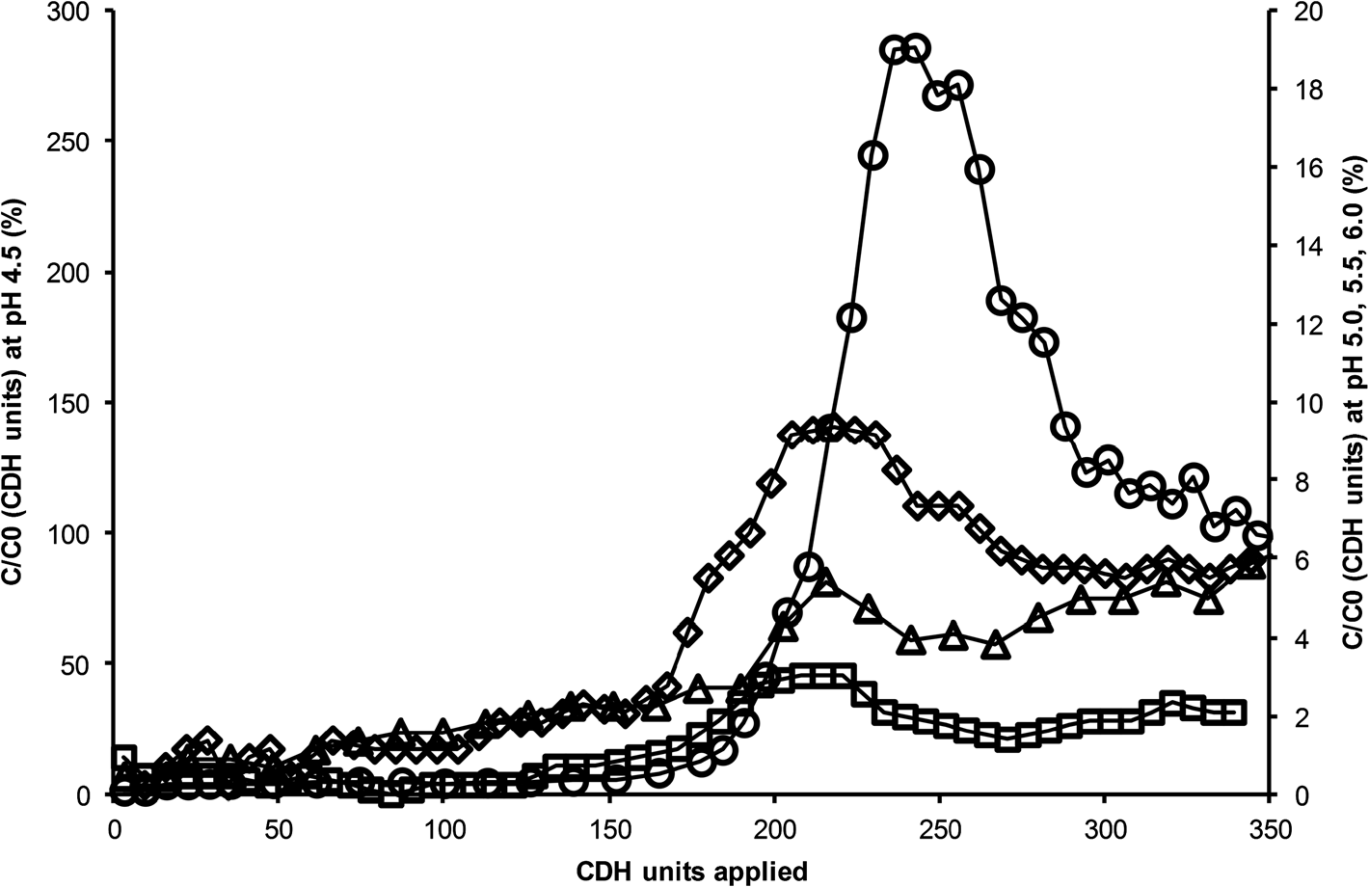
**Breakthrough curves for CDH on Q Sepharose XL.** pH 4.5 (○) on the left axis, pH 5.0 (◊), 5.5 (Δ) and 6.0 (□) on the right axis.

Hence, the CDH breakthrough profiles for various pH values were studied using Q Sepharose XL. A pH of 4.5 is very close to the isoelectric point of the enzyme (as reported to be 4.2 [24]), however the breakthrough stays below 5% until 140 units are applied (Figure 6). After that, enzyme loss increases exponentially reaching 100% breakthrough after 215 units were applied. Even higher c/c_0_ values were obtained meaning that not only no enzyme from the feed solution binds but also that most of the already bound enzyme is washed from the column. When setting the start buffer to a pH of 5.5 or 6.0 the maximum breakthrough is 6% and 3% respectively. Table 1 shows the calculated dynamic binding capacity (defined at 5% breakthrough) for CDH for both columns at the studied pH values. For pH 6.0 dynamic binding capacity could not be determined properly as breakthrough stayed below the critical value of 5%. As can be seen, with the DEAE Sepharose FF column the dynamic binding capacity and therefore the amount of enzyme that should be applied to the column to keep loss at a minimum, is very low (about 60 units) compared to the dynamic binding capacity with the Q Sepharose XL column (about 180 units). As can be seen from Table 1, dynamic binding capacity is rising with increasing pH. This can be explained by a stronger net charge of the enzyme and therefore a better binding of CDH to the column material. In theory, when using pH values closer to the isoelectric point, the binding of other proteins should be less. However, as can be seen from Table 1, this effect is marginal as the specific enzyme activity determined in the elution peak is only a little higher at pH 4.5 (only 160 units were applied as otherwise all enzyme would be lost during sample application and wash) than at the other pH values studied. But, more other proteins compared to CDH bind on the DEAE Sepharose FF column as indicated by the low specific activity of 1 U/mg which is another reason for not choosing this material for purification. Nevertheless, Baminger et al. [24] used a DEAE Sepharose FF column as a capture step with a good purification effect. However, this result could not be obtained during this study (purification fold about 2.3 when applying 50 units).

**Table 1:** **dynamic binding capacity determined at 5% breakthrough and specific enzyme activity in the elute**

**Column**	**pH value**	**Dynamic binding capacity (Units)**	**Specific activity (U/mg)**
Q Sepharose XL	4.5	140	1.6
Q Sepharose XL	5.0	180	1.5
Q Sepharose XL	5.5	210	1.5
Q Sepharose XL	6.0	> 350	1.5
DEAE Sepharose FF	5.0	62	1.0

**Figure 7:**
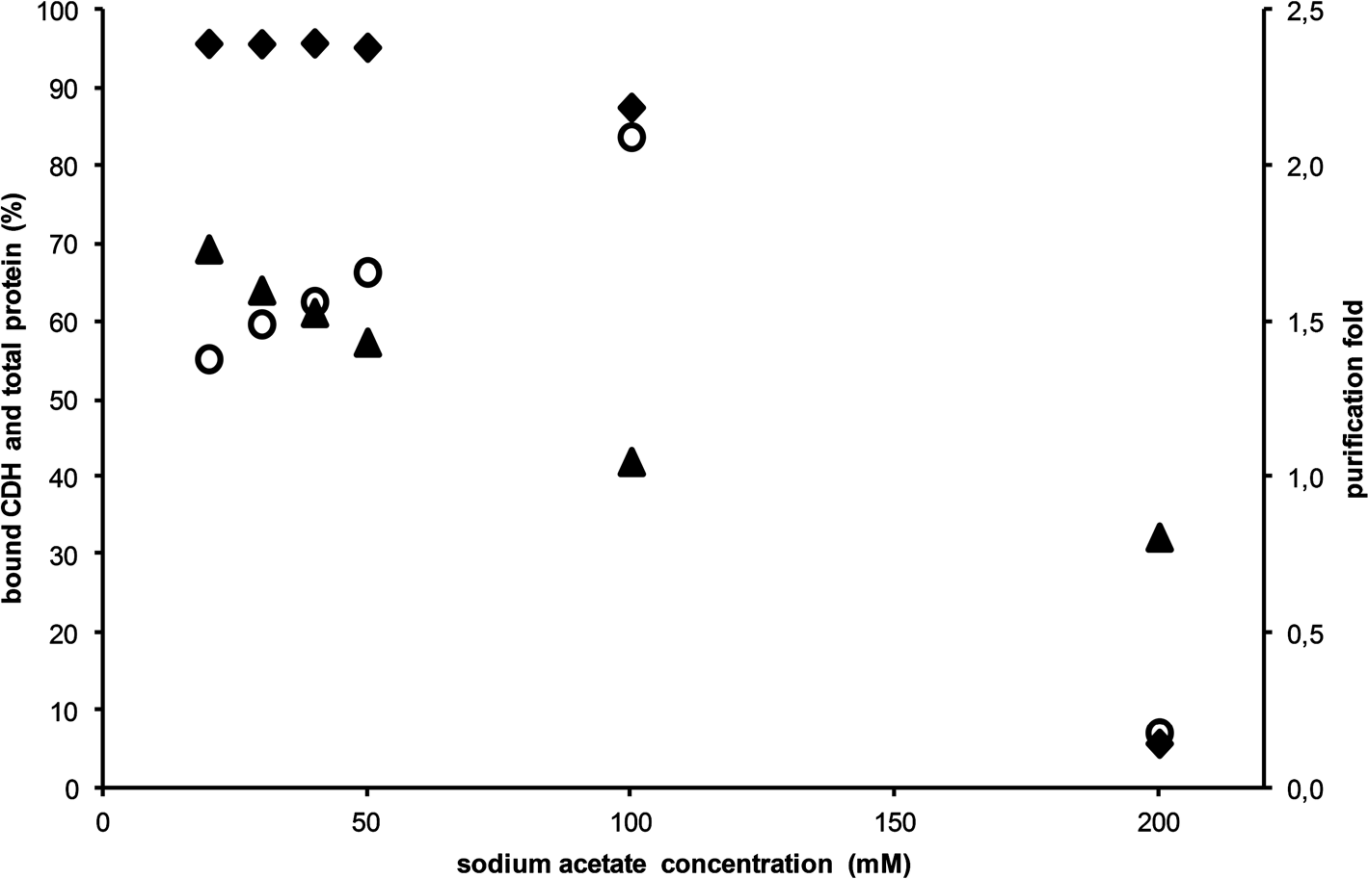
**CDH purification as a function of buffer concentration on Q Sepharose XL.** amount of bound CDH units (♦, left axis), total protein (▲, left axis), purification factor (○, right axis).

Next, ionic strength of the starting buffer was varied from 20 mM to 200 mM sodium acetate (pH 4.5) to determine the maximum possible molarity where CDH still binds but most of the other proteins do not. About 130 units CDH were applied to stay below the previously determined dynamic binding capacity. Three samples were taken from each run and analyzed for CDH activity and protein content during sample application, washing, and elution. As expected, the amount of total bound protein is decreasing with increasing ionic strength (Figure 7). Concurrent, about 95% of the applied CDH is bound to the column at buffer concentrations from 20 to 50 mM. As a consequence, the purification factor is rising. At a sodium acetate concentration of 100 mM only 87.5% of the enzyme is bound. However, the purification is better because simultaneously to some enzyme loss, a comparatively larger amount of other proteins is also not able to bind. Indeed, when the start buffer has a concentration of 200 mM, nearly the entire enzyme is eluted during sample application and washing therefore resulting in a loss of 95%. A loss of about 12.5% as detected at 100 mM was decided to be acceptable. Therefore this ionic strength was used in further studies.

**Figure 8:**
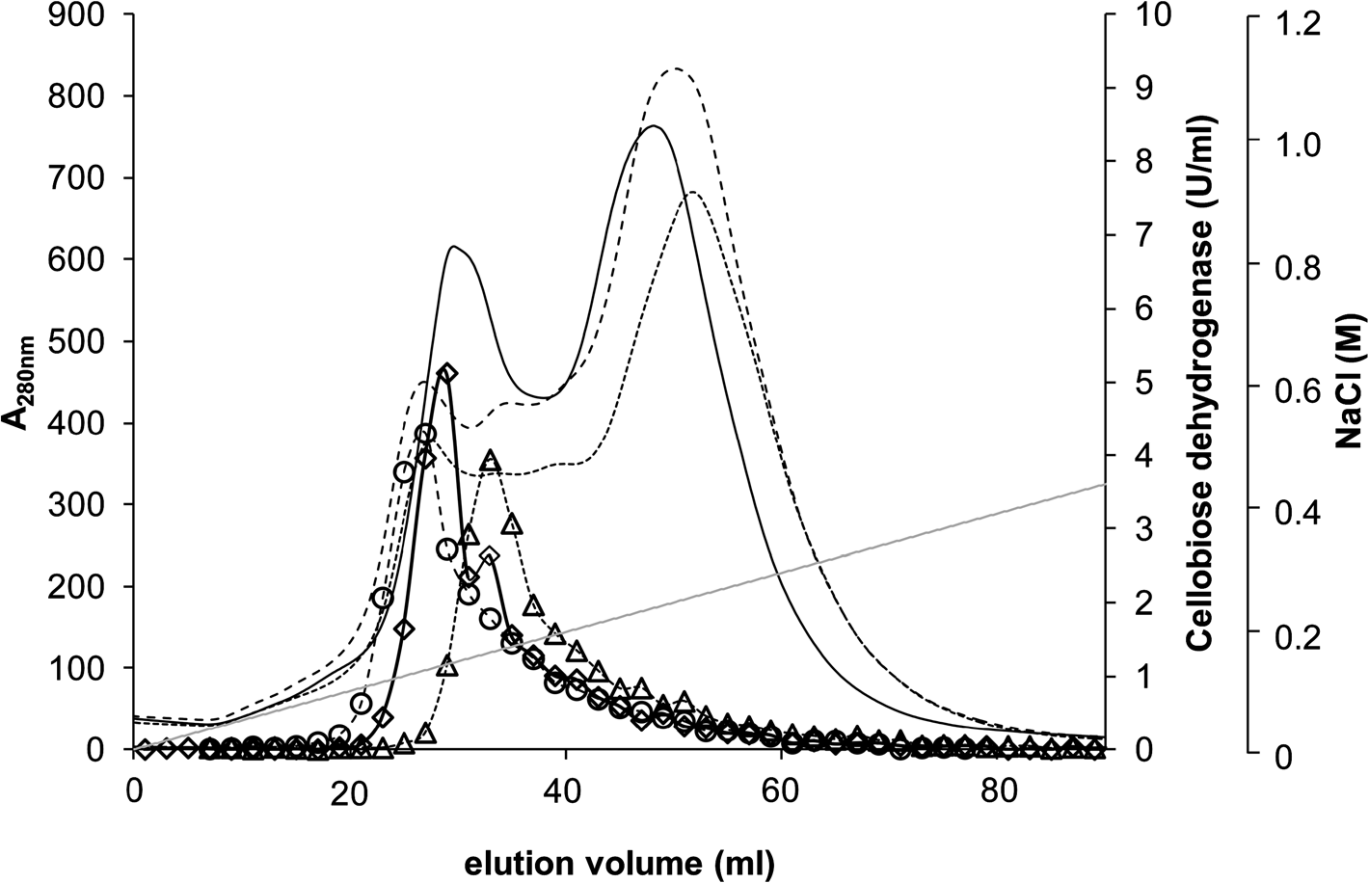
**Elution profiles of CDH on Q Sepharose XL at various pH values.** pH 4.5 (CDH ◊, A_280nm_ straight line), pH 6.0 (CDH ○, A_280nm_ dashed line), pH 8.0 (CDH Δ, A_280nm_ fine dashed line), elution buffer was 1 M NaCl in 100 mM sodium acetate, gradient from 0 to 0.4 M NaCl in 20 CV.

Elution conditions were optimized by first using a linear gradient from 0 to 0.4 M NaCl in 100 mM sodium acetate buffer at various pH values (4.5, 6.0 and 8.0) in 20 column volumes (CV). Two not well resolved peaks were obtained when eluting with the same pH (4.5) as was used during sample application and wash. CDH eluted mainly in the first peak but had a distinct tailing into the second peak (Figure 8). When setting the pH to 6.0, peak separation was better because a number of other proteins were more strongly retained onto the column compared to CDH. Thus, purification factor was rising from 3.3 to 4.7. With further increasing of the pH to 8.0 CDH was also bound more strongly to the column material, i.e. some other proteins eluted first, followed by CDH and more proteins from this complex mixture. The purification factor remained the same (4.7). A pH value of 6.0 was considered to be most suited for elution.

#### Optimization of hydrophobic interaction chromatography

To choose an appropriate hydrophobic interaction medium for the second purification step, the binding properties of several materials was determined by applying 30 units onto the columns and determining breakthrough curves. From the various columns tested, only Phenyl HP was suited for CDH purification as with the other columns CDH binding was weak indicated by high c/c_0_ values right from the beginning of sample application (data not shown). Using Butyl-S FF, Octyl FF and Phenyl FF low sub the entire enzyme was lost during sample application and washing. With the Butyl-FF column some of the enzyme was bound but loss was still high with 70%. Therefore all further experiments were carried out with a Phenyl HP column.

Next, ammonium sulfate concentration (0.5 M, 0.9 M, and 1.1 M) in the starting buffer was varied. Also, the effect of adding NaCl (0.2 M, 2.1 M) was studied. An ammonium sulfate concentration of 0.5 M is too low which could also be seen from the breakthrough profile as most of the enzyme was not bound therefore resulting in a high loss of nearly 70%. Raising the concentration of (NH_4_)_2_SO_4_ to 0.9 M (as was used by Baminger et al. [24] on a Phenyl Resource column) was also not sufficient as most of the target enzyme was eluting during the washing step (loss about 60%). Thus, 1.1 M ammonium sulfate was used as loss was only a third (19%). When adding 0.2 M NaCl to the starting buffer, enzyme loss could be minimized (about 10%). A further increase of NaCl to 2.1 M resulted in an enhanced binding of other proteins which in turn resulted in a worse separation during elution. To gain a certain specific activity more enzyme loss would have to be taken into account.

#### Tandem purification protocol

A method was established that combined IEX and HIC to purify CDH further since enzyme purification using either IEX or HIC was not sufficient. As higher enzyme loading is possible for IEX on Q Sepharose XL, a pH of 5.0 was chosen for the starting buffer (100 mM sodium acetate). Sample solution was diluted 2-fold with starting buffer (resulting in about 20 U/ml) and applied to the column at a speed of 1 ml/min. All further steps were carried out at 5 ml/min. The optimized elution profile includes a linear increase from 0 to 0.15 M NaCl in 8 CV, a hold at 0.15 M NaCl for 4 CV and a stepwise increase to 1 M NaCl (hold 7 CV). After an ammonium sulfate precipitation the supernatant was applied to HIC on a Phenyl HP column (volume: 5 ml). The equilibration buffer contained 1.1 M ammonium sulfate and 0.2 M NaCl in 100 mM sodium acetate buffer pH 5.0. Flow rate was set to 5 ml/min for the entire procedure. Elution was performed by increasing buffer B (50 mM sodium acetate pH 5.0) from 0 to 100% in 8 CV. Table 2 shows the overall purification protocol.

**Table 2:** **Purification of cellobiose dehydrogenase from** ***Sclerotium rolfsii***

**Purification step**	**CDH activity (Units)**	**Total protein (mg)**	**Specific activity (U/mg)**	**Purification fold**	**Yield (%)**
Crude extract	1173	1112	1.1	1.0	100
IEX (Q Sepharose XL)	974	456	2.1	2.0	83
(NH_4_)_2_SO_4_ precipitation	750	398	1.9	1.8	64
HIC (Phenyl HP)	661	56	11.9	11.3	56

However, the obtained enzyme solution after hydrophobic interaction chromatography still contains some other proteins than CDH as could be detected by SDS-PAGE, i.e. the final enzyme solution is not 100% pure. To evaluate if the other proteins have a negative effect on the oxidation of lactose to lactobionic acid (i.e. hydrolysis into glucose and galactose catalyzed by enzymes like α-galactosidase or β-glucosidase which are also produced by the fungus [2]), the enzyme solution was used in a 4.8% lactose solution. For comparison, the crude enzyme extract was used in another experiment.

**Figure 9:**
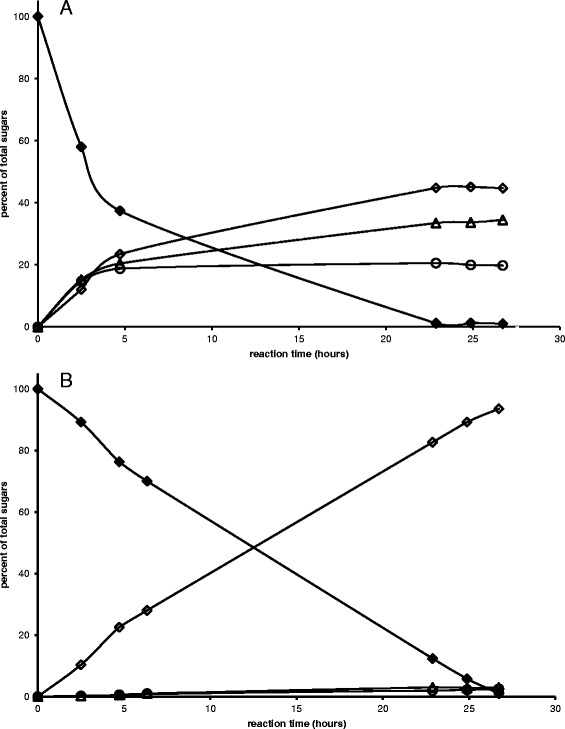
**Oxidation of lactose using crude enzyme extract (A) and purified enzyme (B).** lactobionic acid (◊), glucose (○), galactose (Δ), lactose (♦).

As can be seen clearly from Figure 9A, using unpurified extract leads to high amounts of glucose and galactose caused by α-galactosidases or β-glucosidases as described before. Thus, yield of lactobionic acid was only 45%. Using partial purified enzyme as described in this paper (Figure 9B), only minor amounts of monosaccharides (3% galactose, 2.5% glucose) could be detected and therefore the obtained purity is sufficient for the intended use of CDH. In 27 hours it was possible to obtain a lactobionic acid yield of 94% which is more than twice as much compared to the crude enzyme solution. However, one might also consider using the unpurified extract depending on the exact application, thus saving costs for enzyme purification. Lactobionic acid might further be tested regarding its potential prebiotic effect [11]. The tolerance in humans is already shown [25]. It can further be used as a calcium salt in food applications as a stabilizer. It is also commonly used in the Wisconsin transplantation solution as an organ preservative [26].

### Preservation of purified CDH

Experiments for evaluating two possibilities (freezing and freeze-drying) to preserve CDH were carried out at high enzyme concentration (11,000 U/L) as residual activity is generally higher at higher enzyme concentration [27,28]. Sugars are known to be effective in stabilizing enzymes during freeze-drying therefore acting as lyoprotectants [27,29,30] but no studies on CDH recovery during freeze-drying are known so far.

**Figure 10:**
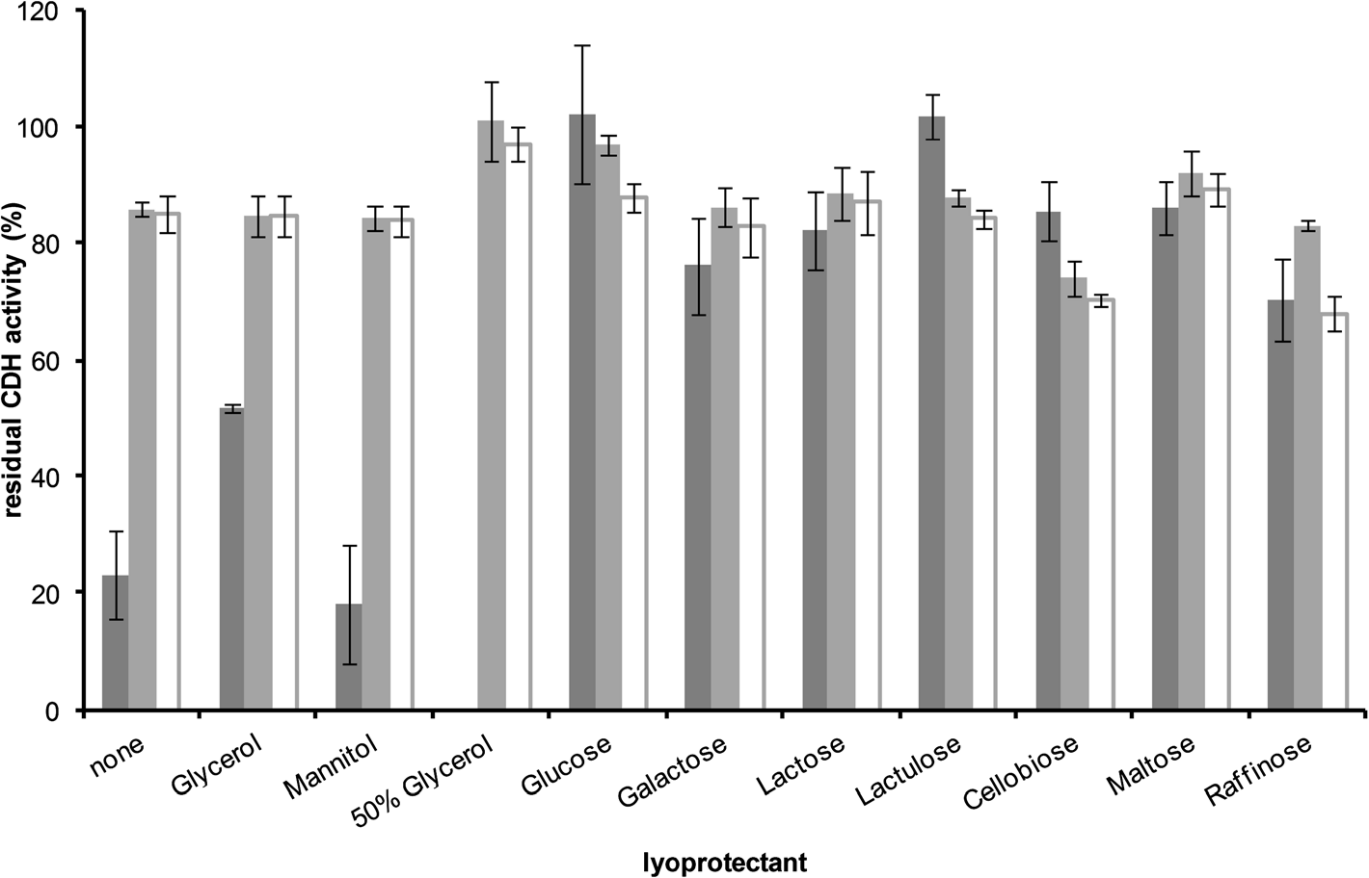
**Preservation of CDH.** The figure shows the residual CDH activity after lyophilisation (dark grey) or freezing after 60 hours (light grey) or 160 hours (white) in the absence (none) or presence of different lyoprotectants (10 μmol/unit), n = 3.

Figure 10 shows the residual CDH activity compared to the sample before freezing or freeze-drying. As can be seen, without using a lyoprotectant most of the CDH is inactivated during freeze-drying leaving a residual activity of only 23%. Glycerol could stabilize the enzyme to some extent while mannitol could not. In general, it was more effective using sugars as stabilizing agents than sugar alcohols. With glucose and lactulose no loss in activity was observed. With the other disaccharides lactose, cellobiose, and maltose over 80% of CDH could be retained while galactose and raffinose are considered to be less effective. However, residual activity was a lot higher compared to the two sugar alcohols tested.

In another series, storage of the enzyme at -20°C was evaluated. In general, a solution of 50% (w/v) glycerol is often used when trying to make enzymes storable. Therefore, as expected, using high glycerol concentrations gave a residual activity of 100% after 60 hours of storage. However, after 160 hours a small decline is observed. Also, glucose seems to be very effective although enzyme stability might not be satisfying as indicated by the relatively high drop in CDH activity after 160 hours compared to stable activity values when using lactose, glycerol, mannitol or no lyoprotectant. On the other hand, cellobiose clearly has a negative effect in enzyme stability during freezing as residual activity values are below the ones when using no additives. Raffinose, similar to glucose, is stabilizing the enzyme at the beginning of the process but with prolonged storage it seems to be less effective and therefore being not suitable.

Generally, freezing at -20°C effects enzyme activity to a much lesser extent than freeze-drying as can be seen from the data when using no further agents. Although it is shown that with adding glucose or lactulose no activity is lost, the process of freeze-drying is obviously more cost and time consuming than simply freezing the solution at -20°C. Additionally, when adding the right amount of glycerol (no effect was observed when using only 10 μmol per unit) it is also possible to retain all of the enzyme activity. It has to be noted that the experiments carried out during this study are only considered to be preliminary. More research is necessary in studying enzyme behavior during storage, for example determining the half-life time.

## Conclusions

To produce cellobiose dehydrogenase from *Sclerotium rolfsii*, it was possible to simplify a rather complex cultivation medium to a basic medium consisting only of α-cellulose, peptone from meat, and trace elements. Additionally, enzyme production was increased by 21%. Purification of the enzyme was studied systematically resulting in a yield of 56%. Although other proteins were detected in the resulting enzyme solution, its purity was considered to be sufficient for oxidizing lactose to lactobionic acid. With freeze-drying of CDH, glucose and lactulose could be identified to be good lyoprotectants. However, freezing at -20°C is preferred for storage as this method being much simpler and residual activity being 100% when using 50% (w/v) glycerol.
